# The Gatekeepers in the Mouse Ophthalmic Artery: Endothelium-Dependent Mechanisms of Cholinergic Vasodilation

**DOI:** 10.1038/srep20322

**Published:** 2016-02-02

**Authors:** Caroline Manicam, Julia Staubitz, Christoph Brochhausen, Franz H. Grus, Norbert Pfeiffer, Adrian Gericke

**Affiliations:** 1 Department of Ophthalmology, University Medical Center of the Johannes Gutenberg University Mainz, Mainz, Germany; 2 Institute of Pathology, University Medical Center of the Johannes Gutenberg University Mainz, Mainz, Germany

## Abstract

Cholinergic regulation of arterial luminal diameter involves intricate network of intercellular communication between the endothelial and smooth muscle cells that is highly dependent on the molecular mediators released by the endothelium. Albeit the well-recognized contribution of nitric oxide (NO) towards vasodilation, the identity of compensatory mechanisms that maintain vasomotor tone when NO synthesis is deranged remain largely unknown in the ophthalmic artery. This is the first study to identify the vasodilatory signalling mechanisms of the ophthalmic artery employing wild type mice. Acetylcholine (ACh)-induced vasodilation was only partially attenuated when NO synthesis was inhibited. Intriguingly, the combined blocking of cytochrome P_450_ oxygenase (CYP450) and lipoxygenase (LOX), as well as CYP450 and gap junctions, abolished vasodilation; demonstrating that the key compensatory mechanisms comprise arachidonic acid metabolites which, work in concert with gap junctions for downstream signal transmission. Furthermore, the voltage-gated potassium ion channel, K_v_1.6, was functionally relevant in mediating vasodilation. Its localization was found exclusively in the smooth muscle. In conclusion, ACh-induced vasodilation of mouse ophthalmic artery is mediated in part by NO and predominantly *via* arachidonic acid metabolites, with active involvement of gap junctions. Particularly, the K_v_1.6 channel represents an attractive therapeutic target in ophthalmopathologies when NO synthesis is compromised.

The magnitude of global visual impairment is estimated to reach an approximate 285 million people at present and this number is expected to escalate as a result of the aging population^1^. Among the many factors that contribute towards vision loss, there is accumulating evidence that emphasizes the dysregulation of ocular blood flow as one of the leading causes of various sight threatening ophthalmopathologies namely, glaucoma, diabetic retinopathy and nonarteritic anterior ischemic optic neuropathy (NAION)^2^,^3^,^4^,^5^,^6^. Modulation of the ocular blood flow is largely attributed to the vascular endothelium, which consists of a group of highly specialized cells that play indispensable physiological roles in the maintenance of vascular tone, especially by the release of various diffusible vasoactive substances. Although nitric oxide (NO) is the common messenger molecule implicated in the vasodilatory responses in ocular blood vessels^7^,^8^, other key mediators released by the endothelium include prostacyclin (PGI_2_) and the endothelium-derived hyperpolarizing factor (EDHF) that play crucial roles in maintaining the hemodynamic balance in ocular vasculature^9^.These mediators can be released by shear stress, autacoids or neurotransmitters from the autonomic nervous system^10^,^11^. One important substance that can act as an autacoid or neurotransmitter of the parasympathetic nervous system and has been shown to induce marked vasodilation in many blood vessels is acetylcholine (ACh). Previous studies reported the presence of nitric oxide synthase (NOS) in the endothelium of ocular arteries and demonstrated its contribution to ACh-induced vasodilation in a wide range of species including in humans^12^,^13^, dogs^14^, rats^15^, pigs^16^, cows^17^, and primates^18^,^19^ On the other hand, it has been shown in different ocular vascular beds that apart from NO, PGI_2_ and EDHFs may also contribute to endothelium-dependent vasodilation^20^,^21^. In recent years, with the use of gene-knockout mice and isoform-selective NOS inhibitors, we demonstrated that endothelium-dependent vasodilation induced by ACh is mediated predominantly by the activation of endothelial NOS (eNOS) in the retinal arterioles^22^. However, in the mouse ophthalmic artery, eNOS mediated only a part of the cholinergic vasodilation response while another, yet unknown, mechanism also substantially contributed towards ACh-induced vasodilation^23^.The mouse ophthalmic artery is a small vessel with an inner diameter between 80 and 150 μm that develops moderate myogenic tone^24^,^25^,^26^. Since the relative contribution of these three mediators to agonist-induced vasodilation varies among vascular beds, species and also the diameter of the blood vessels, we hypothesize that PGI_2_ and EDHFs may be of physiological relevance in the mouse ophthalmic circulation. Therefore, the purpose of the present study was to identify the mechanisms contributing to endothelium-dependent vasodilator responses in the mouse ophthalmic artery using *in vitro* vascular preparations and pharmacological inhibitors. For the first time, this experimental approach allowed for in-depth identification of post-receptor signalling pathways involved in the vasodilatory mechanisms of the mouse ophthalmic artery. Physiologically, the specific modulator molecules and potassium ion channels identified may be potential targets for therapeutic intervention to enhance the circulatory status of the eye in pathologic conditions when NO availability is compromised.

## Results

### Cholinergic vasodilation responses are endothelium-dependent

**Figure 1:**
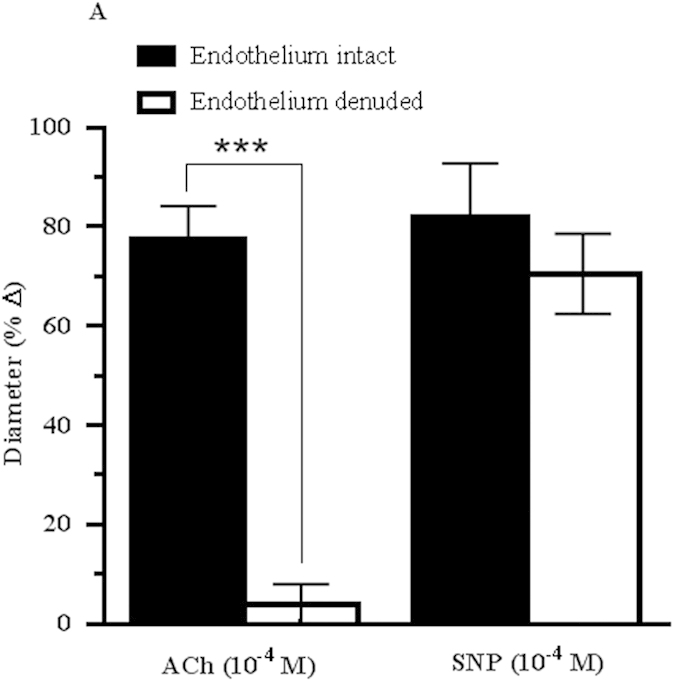
Responses of the wild-type mouse ophthalmic artery before and after removal of endothelium to ACh (10^−4^ M) and to the exogenous NO donor, SNP (10^−4^ M). Vasodilatory responses to ACh were markedly attenuated in the endothelium-denuded vessels, whereas vasodilation responses to SNP were retained in both endothelium-denuded and –intact vessels. Values are expressed as mean ± standard error of the mean (s.e.m) (n = 6 per group; ***P < 0.0001, endothelium-denuded versus endothelium-intact).

To investigate the role of endothelium in mediating cholinergic vasodilation of the OA, preconstricted arteries with intact and denuded endothelium were stimulated with ACh (10^−4^ M). The removal of endothelium in ophthalmic arteries resulted in marked attenuation of the vasodilator response (3.77 ± 4.20%, *P* < 0.0001) compared to the arteries with intact endothelium (77.41 ± 6.78%). Conversely, the vasodilator response in both endothelium-intact (82.00 ± 10.76%) and –denuded (70.53 ± 8.09%) ophthalmic arteries was almost similar following treatment with endothelium-independent, exogenous NO donor, sodium nitroprusside (SNP) (10^−4^ M), indicating that the smooth muscle reactivity remained unaffected after endothelium denudation (Fig. 1).

### Role of NO, and PGI_2_ in ACh-induced vasodilation

**Figure 2:**
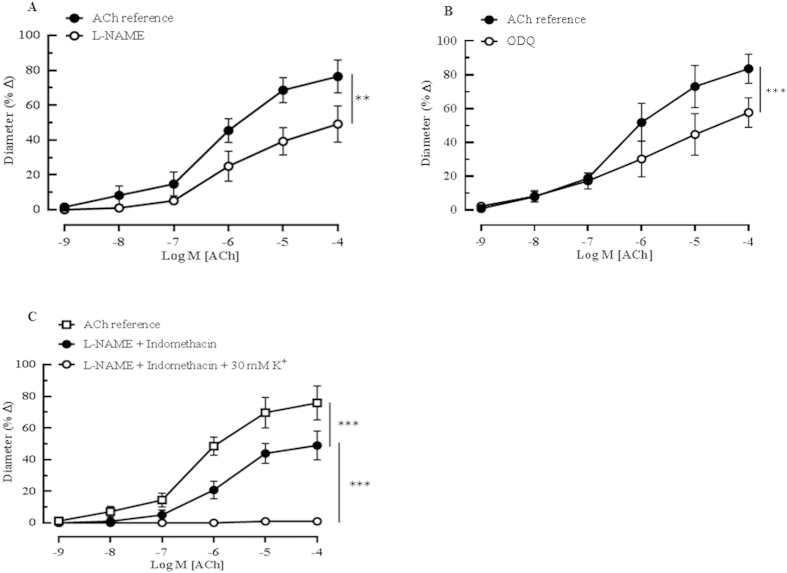
Effect of NOS inhibition on the vasodilatory responses of ophthalmic artery from wild-type mice with intact endothelium. (**A**) The non-subtype-selective NOS inhibitor, L-NAME (10^−4^ M, n = 5) partially attenuated vasodilation to ACh (**P < 0.01, ACh reference *vs* L-NAME). (**B**) The sGC inhibitor, ODQ (10^−4^ M, n = 6) evoked partial yet significant attenuation of the dilatory responses to ACh (***P < 0.001, ACh reference *vs* ODQ). (**C**) The residual dilatory responses in the presence of both L-NAME and indomethacin were abolished by 30 mM of potassium (K^+^) solution (***P < 0.001, ACh reference *vs* L-NAME + Indomethacin; ***P < 0.001, L-NAME + Indomethacin *vs* L-NAME + Indomethacin + KCl). Values are expressed as mean ± s.e.m.

Cumulative administration of ACh (10^−9^–10^−4^M) evoked concentration-dependent vasodilatory responses (76.44 ± 9.45%) that were markedly attenuated (49.18 ± 10.48%, *P* < 0.01) following incubation with the non-isoform-selective NOS inhibitor, L-NAME (10^−4^ M) (Fig. 2A). To test whether the NO receptor, sGC was involved in vasodilation, the ophthalmic arteries were stimulated with ACh (10^−9^–10^−4^ M) before and after addition of the sGC inhibitor, ODQ (10^−5^M). Responses to acetylcholine were markedly reduced (ACh reference: 83.61 ± 8.67% *vs* ODQ: 48.92 ± 8.71%, *P* < 0.001) after ODQ treatment (Fig. 2B), indicative of NO involvement. Conversely, responses to acetylcholine in the ophthalmic arteries cannot be ascribed to PGI_2_ because exposure of the arteries to the non-isoform-selective COX inhibitor, indomethacin (10^−5^M), did not significantly affect the vasodilation (ACh reference: 76.63 ± 5.93% *vs* indomethacin: 69.60 ± 9.84%), as shown in Supplementary Fig. S1. Additionally, combined incubation with L-NAME and indomethacin (48.91 ± 9.01%) did not alter the vasodilatory response compared to inhibition with L-NAME alone (49.18 ± 10.48%) (Fig. 2A,C), suggesting that COX metabolites did not contribute to the cholinergic vasodilation in the mouse ophthalmic artery. However, the residual dilatory response observed after the blocking of both NOS and COX with L-NAME and indomethacin, respectively, was abolished by the addition of 30 mM K^+^ solution (potassium chloride, KCl), as depicted in Fig. 2C. At this concentration, KCl acts as a partial depolarizing agent and antagonizes the action of EDHF. Therefore, this finding suggests the important involvement of EDHF in ACh-mediated vasodilation in this vascular bed.

### Contribution of EDHF-mediated vasodilator responses to ACh

To assess the EDHF mechanisms involved in mediating ACh-induced vasodilation in the mouse OA, various pharmacological agents were employed to inhibit different factors implicated as the putative EDHF. Acetylcholine was previously reported to induce generation of vasoactive amounts of H_2_O_2_ both NOS-dependently and –independently^27^,^28^. To evaluate the contribution of H_2_O_2_ to endothelium-dependent dilatation, responses to ACh before and after incubation with catalase (1000 units/ml) were tested. Catalase, when applied either alone or in combination with both L-NAME and indomethacin, evoked negligible inhibitory effect on vasodilation responses elicited by ACh (Supplementary Fig. S2).

**Figure 3:**
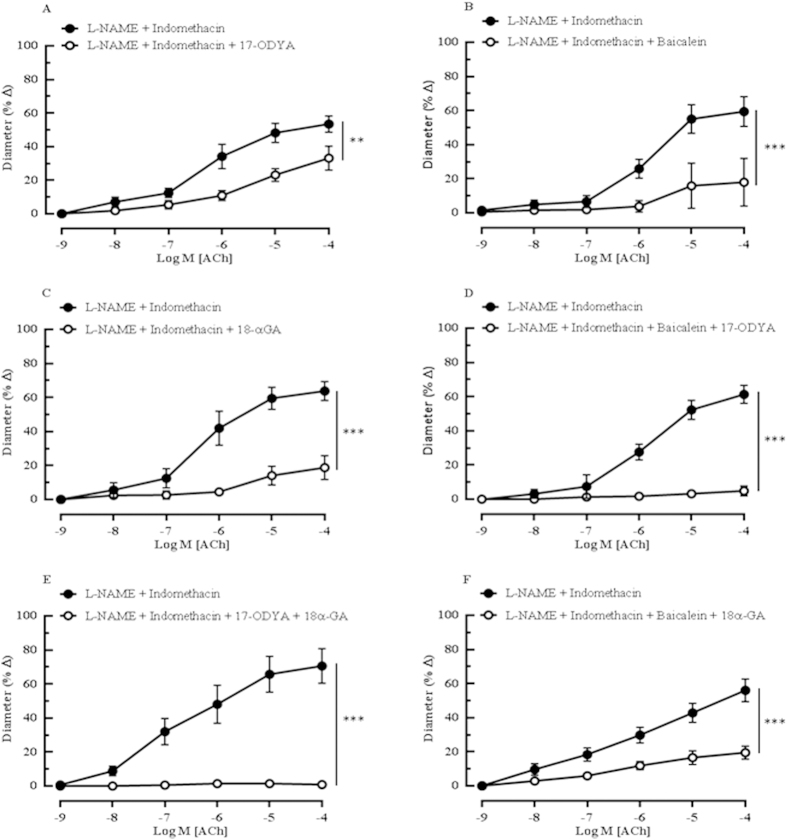
The ACh-evoked vasodilatory responses of ophthalmic artery from wild-type mice mediated by EDHFs. The inhibition of the arachidonic acid metabolites synthesized *via* the (**A**) CYP450 and (**B**) LOX pathways with 17-ODYA and baicalein, respectively, produced significant attenuation of the vasodilation. (**C**) The blocking of gap junctions with 18α-GA caused marked blunting of the dilatory responses. Vasodilation to ACh was almost abolished by the combination blocking with (**D**) 17-ODYA and baicalein and with (**E**) 17-ODYA and 18α-GA. (**F**) Combination blocking with baicalein and 18α-GA caused a partial but significant inhibition of vasodilation. Values are expressed as mean ± s.e.m [n = 5–6 per group; **P < 0.01, ***P < 0.001, L-NAME and Indomethacin *vs* L-NAME and Indomethacin and blocker(s)].

The inhibition of CYP450 and LOX with 17-ODYA and baicalein, respectively, elicited significant blunting of the relaxation (L-NAME and indomethacin: 53.48 ± 4.81% *vs* 17-ODYA: 33.13 ± 7.10%, *P* < 0.01 and L-NAME and indomethacin: 59.36 ± 8.76% *vs* baicalein: 17.98 ± 14.09%, *P* < 0.001, respectively) (Fig. 3A,B). Gap junction inhibitor, 18α-GA, also produced significant inhibitory responses to ACh-mediated dilatation (L-NAME and indomethacin: 63.80 ± 5.55% *vs* 18α-GA: 18.75 ± 7.03%, *P* < 0.001) (Fig. 3C). Interestingly, the combination blocking with 17-ODYA and baicalein almost abolished vasodilation to ACh (L-NAME and indomethacin: 61.28 ± 5.30% *vs* 17-ODYA and baicalein: 4.87 ± 2.86%, *P* < 0.001) (Fig. 3D). In order to investigate which of these two pathways (CYP450 and LOX) may activate the gap junction, ophthalmic arteries were incubated with combinations of 17-ODYA with 18α-GA and baicalein with 18α-GA. The vasodilation elicited by cumulative application of ACh in the presence of L-NAME and indomethacin was abolished by the CYP450 and gap junction blocker combination, as depicted in Fig. 3E (L-NAME and indomethacin: 70.54 ± 10.19% *vs* 17-ODYA and 18α-GA: 0.83 ± 0.55%, *P* < 0.001), whereas combination inhibition of both LOX and gap junction resulted in significant attenuation of vasodilatation (L-NAME and indomethacin: 56.03 ± 6.68% *vs* baicalein and 18α-GA: 19.48 ± 3.84%, *P* < 0.001) (Fig. 3F). These experiments were performed in the presence of both L-NAME and indomethacin to rule out the influence of NOS and COX.

### Effect of potassium ion channel blockers on endothelium-dependent vasodilation

**Figure 4:**
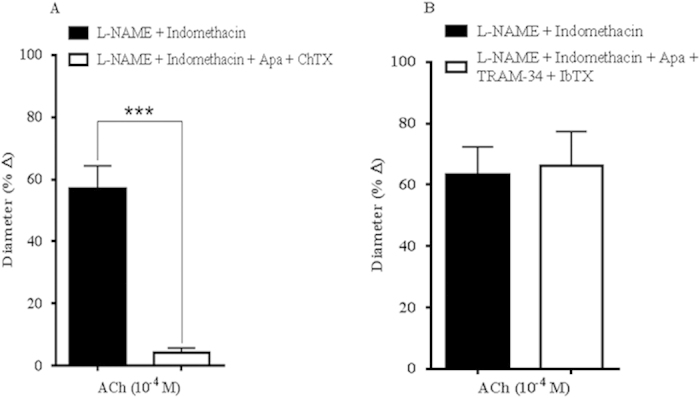
Responses of ophthalmic artery from wild-type mice to ACh-evoked vasodilation in the presence of calcium-activated potassium ion channel blockers. (**A**) Combined blocking with Apa and ChTX elicited total attenuation of endothelium-dependent vasodilation. (**B**) The combination blocking of the KCa channels with their respective specific blockers, Apa, TRAM-34 and IbTX, conferred negligible inhibitory effects on ACh-induced vasodilation. Values are expressed as mean ± s.e.m [n = 5–6 per group; ***P < 0.0001, L-NAME and Indomethacin *vs* L-NAME and Indomethacin and blocker(s)].

To further characterize the EDHF-related dilatation, specifically the contribution fostered by K_Ca_ channels, all three channel subtypes, SK_Ca_, IK_Ca_ and BK_Ca_, were inhibited with combination of Apa and ChTX. Marked inhibition of cholinergic responses was observed with this combination blocking (L-NAME and indomethacin: 56.96 ± 7.40% *vs* Apa and ChTX: 4.07 ± 1.65%, *P* < 0.0001), as shown in Fig. 4A. To further validate this finding, each K_Ca_ channel subtype was blocked in combination with their respective, highly specific inhibitors consisting of Apa for SK_Ca_, TRAM-34 for IK_Ca_ and IbTX for BK_Ca._ Remarkably, this combination blocking demonstrated that the vasodilator response to ACh remained unchanged (L-NAME and indomethacin: 63.26 ± 9.07% *vs* Apa and TRAM-34 and IbTX: 66.06 ± 11.34%, *P* > 0.05) (Fig. 4B). Each of these three K_Ca_ channels was inhibited individually with Apa, TRAM-34, IbTX and ChTX and our findings showed that only the inhibition with ChTX displayed significant attenuation of the vasodilation (Supplementary Fig. S3), while the other blockers comprising of Apa, TRAM-34 and IbTX did not contribute to significant blunting of vasodilation in the OA (Supplementary Fig. S4- S6, respectively). Based on these results, we suspected that ChTX, which had also been reported to block several K_v_ channel subtypes, blocked one or more of the K_v_ channel subunits in the mouse ophthalmic artery.

**Figure 5:**
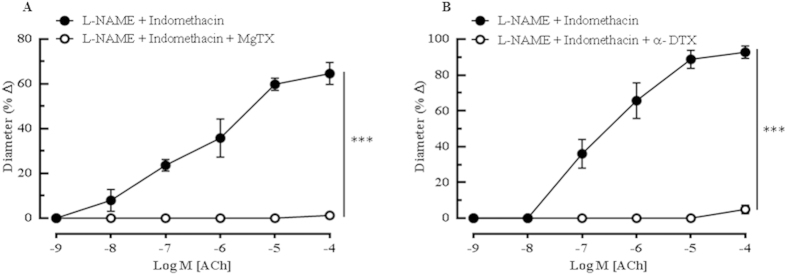
Specific inhibition of the K_v_ channel family in the ophthalmic artery of wild type mice indicated an active participation of this channel subtype in mediating the agonist-evoked vasodilatory mechanisms. (**A**) Blocking with MgTXalmost abolished dilatory responses of the arteries. (**B**)The inhibition of K_v_1.1, K_v_1.2 and K_v_1.6 channels with α-DTX demonstrated a significantabolishment of vasodilation. Values are expressed as mean ± s.e.m [n = 5–6 per group; ***P < 0.001, L-NAME and Indomethacin *vs* L-NAME and Indomethacin and blocker(s)]. Absence of error bar indicates that the SEM was less than the size of the symbol.

Hence, to confirm this hypothesis, a number of agents that block K_v_ channels were employed to assess the nature of the K_v_ channel involved in mediating ACh-elicited vasodilation. In order to determine which of these ChTX-sensitive K_v_ channel subtype(s) is particularly involved in mediating the ACh-induced relaxations, vessels were incubated with MgTX, a selective blocker of the K_v_1.3 and K_v_1.6 channels in the presence of NOS and COX inhibitors. Incubation with MgTX completely blunted ACh-induced dilations (L-NAME and indomethacin: 64.67 ± 4.93% *vs* MgTX: 1.25 ± 1.25%, *P* < 0.001) (Fig. 5A), underscoring the probable role(s) of ChTX- and MgTX-sensitive K_v_1.3 and K_v_1.6 channels in the mouse ophthalmic arteries. Following incubation with MTX, psora-4 and β-DTX which, specifically blocks the K_v_1.2, K_v_1.3 and combinations of K_v_1.1 and K_v_1.2 channels, respectively, the ACh -induced vasodilatation remained unaffected ((Supplementary Figs S7–9) In contrast, incubation with α-DTX, a potent blocker of K_v_1.1, K_v_1.2 and K_v_1.6 channels evoked complete attenuation of the vasodilatory responses to ACh, as demonstrated in Fig. 5B (L-NAME and indomethacin: 92.73 ± 3.48% *vs* α-DTX: 4.96 ± 2.34%, *P* < 0.001). Taken together, the results of the K_v_ channel inhibitions is attributed to the involvement of the K_v_1.6 channel that is sensitive to the blocking effects of ChTX, MgTX as well as α-DTX.

**Figure 6:**
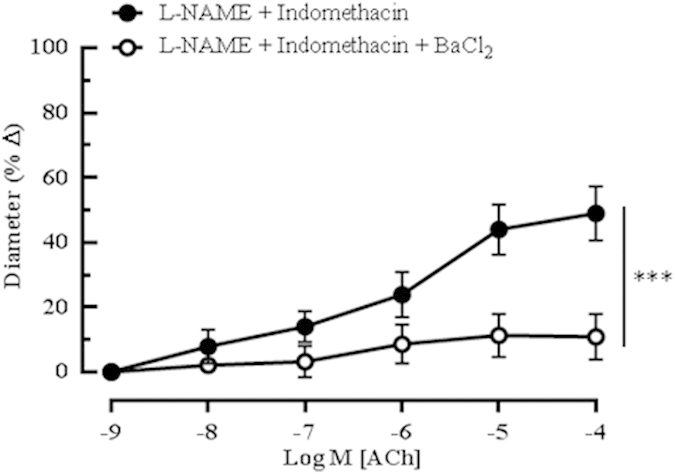
Cholinergic vasodilation of the mouse ophthalmic artery in the presence of Kir channels blocker. The inhibition of Kir channels with BaCl_2_ caused a significant inhibition of dilation. All experiments were carried out in the presence of both NOS and COX inhibitors. Values are expressed as mean ± s.e.m (n = 5 per group; ***P < 0.001, L-NAME and Indomethacin *vs* L-NAME and Indomethacin and blocker).

Next, to evaluate the potential functional relevance of other potassium channels, arteries were treated with blockers of Kir and K_ATP_ channels, and Na^+^/K^+^-ATPase. Neither glibenclamide nor ouabain had any significant effect on the ACh-mediated vasodilation ((Supplementary Figs S10–11)), indicating the null involvement of the K_ATP_ channel and Na^+^/K^+^-ATPase, respectively. Conversely, Fig. 6 shows that BaCl_2_, a blocker of Kir channels, caused 38.07% inhibition (*P* < 0.001) of the ACh-elicited vasodilation in the presence of L-NAME and indomethacin (L-NAME and indomethacin: 48.64 ± 8.35%).

### Localization of the K_v_1.6 channel in the mouse ophthalmic artery

**Figure 7:**
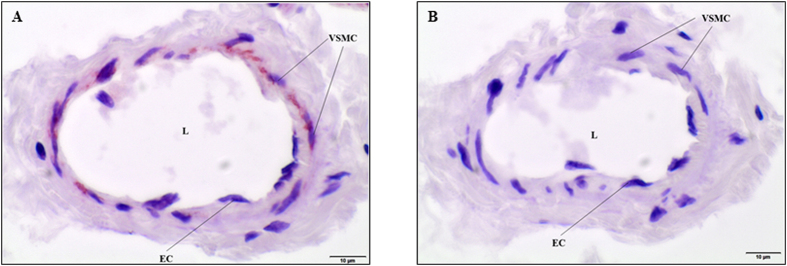
Photomicrographs representing the transverse cryosections of the mouse ophthalmic artery for K_v_1.6 channel immunolocalization. (**A**) The expression of the K_v_1.6 channel is prominent in the smooth muscle cell layer compared to the endothelial cells. (**B**) The negative control demonstrates no staining in the absence of the primary antibody. EC, Endothelial cell; VSMC, Vascular smooth muscle cell; L, Lumen. Scale bars indicate 10 μm at 600× magnification.

The K_v_1.6 channel plays a central role in the regulation of the ophthalmic blood flow as demonstrated by the functional experiments in the present study. In order to determine the localization of this K_v_ channel subtype in the ophthalmic artery, immunostaining was carried out on the sagittal cryosections of ophthalmic artery. Localization of K_v_1.6 was particularly restricted to the vascular smooth muscle cell layer but no expression was observed in the endothelial cells, as shown in Fig. 7A. The negative control of the same tissue in which the primary antibody was omitted, was not stained (Fig. 7B).

## Discussion

This is the first functional study reporting on the EDHF mechanisms mediating agonist-induced vasodilator response in the mouse ophthalmic artery. There are several key findings, including some novel aspects, emerging from the current study. First, in addition to the well-established observations of the role of endothelium in various vascular beds and species, we endeavoured to investigate the role of endothelium in vasodilator response to ACh particularly in the mouse ophthalmic artery. The use of ACh instead of other agonists e.g. bradykinin is advocated in this study to circumvent desensitization of the endothelial receptors due to tachyphylaxis^10^. Moreover, our previous study has clearly demonstrated that in the murine ophthalmic artery, endothelium-dependent vasodilator responses were mediated by the M_3_ muscarinic ACh receptor^25^,^26^. Mechanical denudation of endothelium abolished ACh-induced vasorelaxation, thereby demonstrating that the vascular endothelium plays an obligatory role in the cholinergic vasodilation of mouse ophthalmic artery. Our results also showed that the endothelium-dependent responses were partially mediated by a NOS- and sGC-dependent mechanism, supporting the involvement of NO. These findings are in contrast to the vasodilatory mechanism in the human ophthalmic artery, where the ACh- induced dilation is mediated predominantly by the NO pathway^12^. Conversely, the involvement of PGI_2_ was discounted in the mouse ophthalmic artery because indomethacin exerted no inhibitory effect on the dilatory responses to ACh. These results imply that prostanoid-dependent signalling pathway do not account for the ACh-mediated vasodilatory response in the mouse ophthalmic artery.

Secondly, the predominant involvement of EDHF accounts for the residual dilatory response observed in the mouse ophthalmic artery in the presence of both L-NAME and indomethacin, whereby the abolishment of dilation by concomitant addition of depolarizing concentration of potassium solution was observed^29^. It is widely recognized that the EDHF phenomenon evokes vasodilatation in the presence of COX and NOS inhibitors^30^,^31^ and its physiologic influence is deemed more prominent as the vessel diameter decreases. Since the smaller vessels have fundamental roles in vascular resistance, EDHF is suggested to be of major importance in the blood flow control in these vessels^32^,^33^. Consistent with this possibility, the involvement of these different factors implicated as EDHFs was tested and we found that endothelium-dependent vasodilation in the ophthalmic vasculature was mediated in part by CYP450 and predominantly by LOX metabolites, with a major involvement of the gap junctions. While the individual blockade of CYP450 and LOX only partially reduced vasodilation responses, combined blockade of CYP450 and LOX virtually abolished vasodilation suggesting that metabolites of both enzymes almost exclusively contribute to the EDHF-mediated responses in this vascular bed. The CYP450 pathway appears not to be completely dependent on the gap junctions because combined blockade of both CYP450 and gap junctions only resulted in additive response to the individual inhibitions. It is becoming increasingly well recognized that the arachidonic acid metabolites generated *via* the CYP450 pathway, most likely the four epoxyecosatrienoic acid regioisomers (EETs), have been implicated in the augmentation of gap junctional communications and to regulate active communications between endothelial cells^34^,^35^,^36^. This is especially relevant because EETs are highly lipophilic transferable factors that cannot pass through the gap junctions, which comprise of aqueous pores, but rather may act as modulators to hyperpolarize the VSMC *via* the gap junctions^37^,^38^,^39^.

In contrast to the CYP450-mediated signalling, the LOX signalling mechanism(s) seemed to be highly dependent on the gap junctions, through which the downstream signals and/or molecule(s) that dilate the VSMC are transmitted, since combined blockade of LOX and gap junctions did not result in any further attenuation of the response. However, it should be remarked that the molecular weights of LOX- derived metabolites, namely 15-hydroxy-11, 12-epoxyeicosatrienoic acid (HEETA) and 11, 12, 15-trihydroxyeicosatrienoic acid (THETA,) are large and since the aqueous central pore of the gap junctions can only permit the passage of molecules < 1 kDa, it is unlikely that LOX and/or its metabolites are transferable *via* this channel to hyperpolarize the VSMC^40^,^41^,^42^. However, a plausible explanation can be that arachidonic acid metabolites generated *via* the LOX pathway may act as autocrine or intracellular modulators of gap junctions, as was previously proposed in the rat middle cerebral artery and rabbit arteries, where LOX metabolites directly stimulated the SK_Ca_ channels, instead of the gap junctions as observed in our study, to hyperpolarize the VSMC^43^,^44^,^45^. The murine LOX share a highly conserved sequence similarity with the human’s based on the phylogenetic classification and they belong to the same 12/15-LOX subfamily^46^. Intriguingly, 12/15-LOX was found to be associated with key regulation roles in pathologies of the central nervous system such as Parkinson’s disease and Alzheimer’s^47^,^48^. Looking at the pivotal roles of the LOX- derived metabolites in human pathologies and the high similarity between both mouse and human, these findings broadens the use of murine models for further in-depth investigations of the molecular mechanisms of LOX-related pathway in the next studies.

Despite the rapid progress made in the past decade in elucidating the physiological roles of the arachidonic acid metabolites in various biological systems, many important questions still remain unanswered. For example, in our study, the existence of putative receptor(s) of the downstream signalling cascade of CYP450, especially for the EETs, require further investigation to extend our current hypothesis beyond the present findings. Likewise, studies involving the CYP450 metabolites in cardioprotection are also seeking to identify the precise molecular receptor(s) target(s) of EETs for potential development of new therapeutic strategies^39^,^49^. Additionally, it is important to define the precise identity of the arachidonic acid metabolites generated *via* the CYP450 and LOX mechanisms responsible for the observed vasodilatory phenomenon, as emphasized by Thollon *et al.*^50^.

Thirdly, our data strongly suggest the important involvement of the Kir and K_v_1.6 channels in mediating endothelium-dependent dilation to ACh. Previous studies have shown that K^+^ released from the endothelium can act as an EDHF by activating K_Ca_ and stimulating Na^+^/K^+^-ATPase and Kir channel in guinea pig choroidal arterioles and rat hepatic arteries, respectively^51^,^52^. Therefore, we examined the possible role of potassium channels in endothelium-dependent vasodilation of the mouse ophthalmic artery. Consistent with the finding that K_ATP_ channels are usually not involved in EDHF-mediated vasodilation^53^, our results indicated that the inhibitory effect of glibenclamide on K_ATP_ channel had negligible influence on the vasodilatation of mouse ophthalmic artery induced by ACh. The application of ouabain also failed to inhibit dilation, suggesting the lack of Na^+^/K^+^-ATPase involvement in mediating responses to ACh. However, it is of interest that the blockade of Kir channels caused significant attenuation of vasodilation in the ophthalmic artery. The Kir, channel localized on the SMCs, is one the major targets of external K^+^ions, which activate the channel conductance to lower intracellular Ca^2+^ and leads to vasodilatation^54^,^55^.

Accumulating evidences imply that the action of EDHF is generally inhibited by combination blockade of the SK_Ca_ with Apa and, IK_Ca_ and BK_Ca_ with ChTX^32^,^56^. Correspondingly, our study demonstrated that the combined inhibition with these blockers virtually abolished cholinergic responses in the mouse ophthalmic artery. However, it is important to highlight here that the combined blockade of all three K_Ca_ channels with their respective specific blockers, and not ChTX, had no significant effect on the ACh-mediated vasodilatory responses in the current study as hypothesized. Of note, an interesting phenomenon was observed in mice where the expression of IK_Ca_ and SK_Ca_ in the endothelial cells was relatively low as the size of the artery decreased^57^ which, was in sharp contrast to the increased expression of both channel subtypes in the rat artery as the vessel size decreased^58^,^59^. On the basis of our results, these observations support the hypothesis of our study that the expression of the K_Ca_ channels in the mouse ophthalmic artery may be low or null and are unlikely to account for the attenuation of the vasodilation when blocked with Apa and ChTX. Taken together, this confounding finding can be extrapolated to the multi-channel blocking properties of ChTX, which not only blocks the IK_Ca_ and BK_Ca_ channels, but also inhibits the *Shaker*-related voltage-gated K^+^ channels K_v_1.1, 1.2, 1.3, and 1.6 with high affinity^60^,^61^.

**Figure 8:**
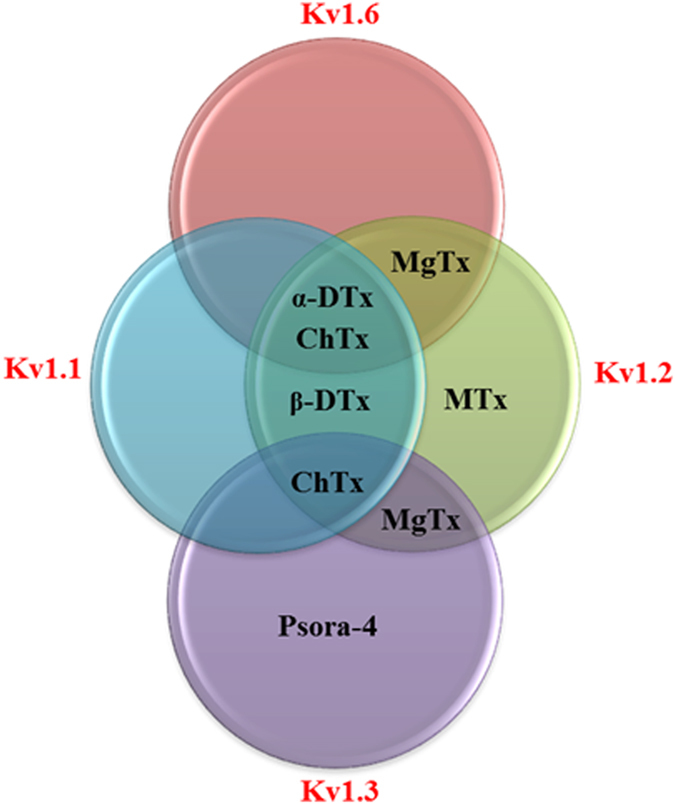
Venn diagram representing the various pharmacological blockers employed in this study to dissect the contribution of the K_v_ channels to ACh-induced vasodilation of the mouse ophthalmic artery. There are overlaps in the affinity of the blockers for one or more K_v_ channels. MgTX, Margatoxin; MTX, Maurotoxin; ChTX, Charybdotoxin; α-DTX, alpha Dendrotoxin; β- DTX, beta Dendrotoxin; Psora-4, [5-(4-Phenylbutoxy)psoralen].

An ongoing, unresolved restriction to study the post- receptor mechanisms is the use of most characterized pharmacological blockers and inhibitors with unspecific nature that may be affecting another alternative EDHF signalling cascade with similar affinity, as demonstrated in the current study. Therefore, to confirm the involvement of the K_v_ channels and in particular to dissect which of these is/are involved in the vasodilation of the mouse ophthalmic artery, several highly specific K_v_ channel inhibitors were employed. Complete attenuation in vasodilation was observed in the presence of MgTX and α- DTX. MgTX is widely used as a potent inhibitor of the K_v_1.3 in ion channel investigations^62^. However, a recent study by Bartok *et al.* provided critical evidence that MgTX is not a highly specific K_v_1.3 inhibitor as had been assumed in many previous studies^63^ and this toxin has also been shown to inhibit other K_v_ channels, namely K_v_1.1, 1.2 and 1.6, with high potency^61^,^64^,^65^,^66^,^67^. Our results support a possible participation of other channel subtype(s) considering the potential overlap in blocking selectivity exhibited by ChTX and MgTX, as summarized in the Venn diagram (Fig. 8). Therefore, with the use of several other toxins, this study unravelled that the K_v_1.6 channel is functionally relevant in mediating vasodilatory responses. Additionally, immunostaining confirmed the localization of this voltage-gated channel subtype in the VSMC of the mouse ophthalmic artery.

**Figure 9:**
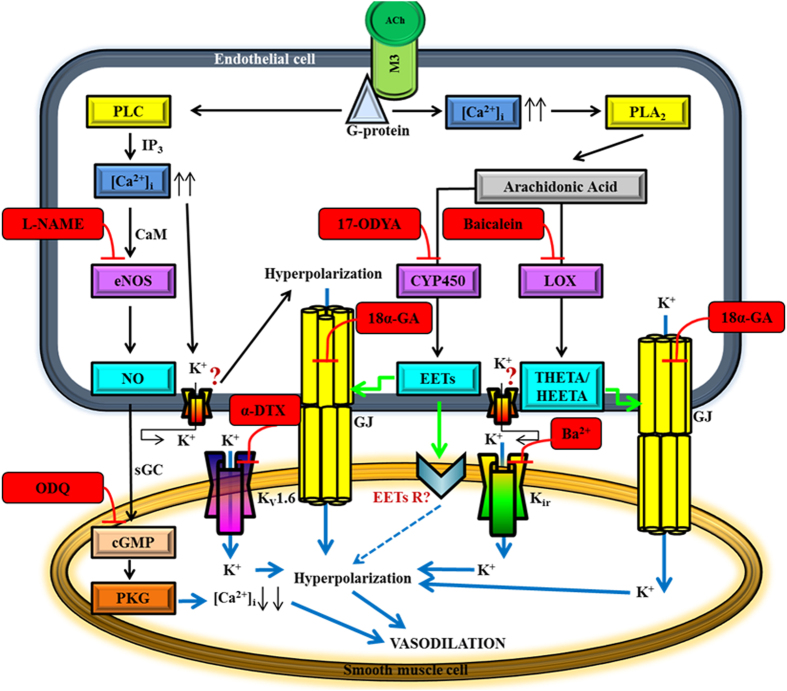
The proposed hypothetical endothelial cell-dependent signalling pathways of conducted vasodilation in response to ACh in the mouse ophthalmic artery. *The first proposed mechanism involves the NO*/*cGMP pathway:* Activation of the endothelial M3 receptor by ACh induces an influx of [Ca^2+^]_i_. Following interaction with CaM (calmodulin), Ca^2+^ activates eNOS and release of NO. NO causes relaxation by interacting with the haem group of the enzyme, sGC, which then mediates the formation of cyclic guanosine monophosphate (cGMP) and activation of protein kinase G (PKG) that relaxes the VSMC. *The second vasodilator mechanism is via the arachidonic acid (AA) metabolites synthesized through the CYP450 oxygenase pathway:* The increase in [Ca^2+^]_i_ elicits translocation of phospholipase A2 (PLA_2_) to the membrane and its major hydrolysis product is AA, which can be metabolized by CYP450 oxygenase to EETs. The EETs function as messenger molecules that modulate gap junctions (GJ) to spread the conductance to the VSMC. EETs may also directly activate a channel/receptor on the VSMC to hyperpolarize and dilate the vessel. *The third signalling pathway involves the AA metabolites generated via the LOX pathway:* It is hypothesized that LOX and/or its metabolites, namely THETA and HEETA do not pass the GJ as EDHF *per se* but activate GJ to hyperpolarize the VSMC. *The forth key players are the gap junctions. The fifth proposed mechanism involves the active participation of the Kir and K*_*v*_*1.6 channels:* It is hypothesized that both Kir and K_v_1.6 channels on the VSMC are activated by the increase in extracellular K^+^ resulting in hyperpolarization and vasodilation. The precise identity of the putative channel(s) on the endothelial cells that is activated and opened for K^+^ efflux for hyperpolarization to occur is unknown. Question marks represent unknown receptors that are yet to be identified. Blockers and inhibitors are indicated in red boxes. Green arrows indicate the activation of the gap junctions and downstream receptor(s). Blue solid arrows show potential pathways for transfer of hyperpolarization from the endothelium to the smooth muscle cells. Blue quadrangular point arrow indicates the hypothesized transfer of hyperpolarization *via* an unknown receptor on the VSMC.

The novelty of the present investigation lies in the identification of the K_v_1.6 channel in mediating vasodilation that has never been reported hitherto. It is well recognized that the altered K_v_1.6 channel expression is associated with neurodegenerative diseases such as Amyotrophic Lateral Sclerosis (ALS) that affects the duration of action potential of motor neurons^68^. On the other hand, study by Carrisoza-Gaytan *et al.* emphasized the importance of the K_v_1.6 channel in K^+^ reabsorption in the thick ascending limb of the rat nephron^69^. This channel is also implicated in the pulmonary artery smooth muscle cells as one of the crucial hypoxia-sensitive K_v_ channels that regulate membrane potential and intracellular Ca^2+^ homeostasis during hypoxia^70^,^71^,^72^. As our understanding of the K_v_ channels continues to evolve, it is therefore tempting to conjecture that the specific identification of the K_v_1.6 channel in this study may represent an innovative molecular target in the ophthalmic circulation to enhance vasodilation in conditions of channelopathy, albeit the exact function of this channel subtype in the ophthalmic circulation warrants further investigation. Our current investigation provides a plausible hypothesis as to how ACh-induced vasodilatation may occur in the mouse ophthalmic artery and based on our results, the hypothesized signalling pathways involved in the vasodilator mechanisms are as depicted in Fig. 9.

In conclusion, the hallmark of this study was the identification of the major signalling cascades that mediate endothelium-dependent vasodilation in the mouse ophthalmic artery which, were previously uncharacterized. Although the findings emerging from this experimental study do not fully account for the precise molecular mechanisms underlying the observed vasodilation *in vitro*, the current elucidation of EDHF mechanisms in mouse ophthalmic artery assigns a pivotal platform for the use of mice to further explore the functional relevance of specific CYP450 and LOX metabolites in mediating ACh-induced vasodilation, as well as the existence of a potential putative, ‘novel’ receptor on the endothelial cells that mediates the efflux of K^+^ remains to be determined in this vascular bed. This study also addressed the potential therapeutic target(s) for future translational applications in human ocular diseases. It will be interesting to determine whether the contribution of the specific potassium ion channels outlined here in the mice ophthalmic artery could also play similar roles in the human ophthalmic circulation, particularly in pathological conditions when NO synthesis is impaired.

## Materials and Methods

### Experimental animals

This study was approved by the Animal Care Committee of Rhineland-Palatinate, Germany, and animal care conformed to the institutional guidelines and ‘The Association for Research in Vision and Ophthalmology’ (ARVO) statement for the use of animals in ophthalmic and vision research. Mice were treated according to the EU Directive 2010/63/EU for animal experiments. Male C57BL/6J mice (The Jackson Laboratory, Bar Harbour, ME, USA) aged 3 to 7 months old were used for the experiments. Animals were housed under standard conditions (temperature 23 ± 2 °C, humidity range 55 ± 10% and 12 h light/dark cycles), and had access to standard mouse chow and water *ad libitum*.

### Drugs

The following drugs were used in this experiment: *N*^ω^-nitro L-arginine methyl ester (L-NAME), indomethacin, acetylcholine hydrochloride (ACh), phenylephrine, 1H-(1, 2, 4) oxadiazole (4, 3-alpha) quinoxaline-1-one (ODQ), catalase, baicalein 18 alpha-glycyrrhetinic acid (18α-GA), ouabain, glibenclamide, barium chloride (BaCl_2_), and psora-4 [5-(4-Phenylbutoxy)psoralen] (all purchased from Sigma-Aldrich Chemie GmbH, Steinheim, Germany), 17-octadecynoic acid (17-ODYA) and 1-[(2-chlorophenyl) Fdiphenylmethyl]-1H-pyrazole (TRAM-34) (Tocris Bioscience, Bristol, UK), iberiotoxin (IbTX), charybdotoxin (ChTX) and apamin (AnaSpec Inc., Fremont, CA, USA), margatoxin (MgTX), maurotoxin (MTX), α- and β-dendrotoxin (α- and β- DTX) (Alomone Labs, Jerusalem, Israel). Indomethacin, ODQ, 17-ODYA, baicalein, glibenclamide and TRAM-34 were dissolved in dimethyl sulfoxide (DMSO). DMSO at ≤ 0.2% (v/v) did not influence vascular reactivity to agonists and antagonists tested, as described elsewhere^73^. 18α-GA was dissolved in chloroform: ethanol (2:3) according to the manufacturer’s instructions and this solvent mixture did not affect the vasoreactivity (personal observation). Apamin, charybdotoxin, iberiotoxin and L-NAME were dissolved in phosphate buffer saline (PBS), whereas all other drugs were dissolved in distilled water.

### Vascular preparation and reactivity studies

Mice were sacrificed by CO_2_ inhalation, and the eyes were rapidly removed and placed in cold Krebs-Henseleit buffer composed of 118.3 mM NaCl, 4.7 mM KCl, 2.5 mM CaCl_2_, 1.2 mM MgSO_4_, 1.2 mM KH_2_PO_4_, 25 mM NaHCO_3_, 11 mM glucose (Carl Roth GmbH, Karlsruhe, Germany). The ophthalmic arteries were carefully isolated and cleaned of surrounding connective tissues using fine-point tweezers under a dissecting microscope. Arterial segments were then placed in an organ bath with ice-cold Krebs-Henseleit buffer, cannulated onto two glass micropipettes and secured with 10–0 nylon monofilament suture. Vessels were pressurized *via* these micropipettes to 50 mm Hg under no-flow conditions using two reservoirs filled with Krebs-Henseleit buffer. The ophthalmic artery was equilibrated for 30–40 minutes before the commencement of the experiments. During this equilibration period, the vessels developed a stable spontaneous myogenic tone by constricting to ~86 to 81% of the initial arterial luminal diameter measured immediately after pressurization to 50 mmHg, as described elsewhere^24^. Video sequences were captured to a personal computer using a video camera mounted on an inverted microscope for off-line analysis. The organ bath was continuously circulated with Krebs solution maintained at 37 °C and pH 7.4 and, aerated with 95% O_2_ and 5% CO_2_. A minimum 50% vasoconstriction from the resting diameter in response to 100 mM K^+^ solution was used as a criterion to assess vessel viability^26^.

In some experiments, the endothelium was mechanically removed by rubbing the luminal surface of the arteries with a human hair, as described previously^26^. Next, arteries were preconstricted to 70–50% of the initial vessel diameter by titrating the α_1_-adrenoceptor agonist phenylephrine, and concentration-response curves to ACh (10^−9^–10^−4^ M) was obtained by cumulative application of ACh to the circulating bath solution. The pre-treatment of the arteries with L-NAME slightly constricted the vessels and in this circumstance, the phenylephrine concentration was adjusted to reach a similar preconstriction level in all experiments. All reported drug concentrations refer to final molar concentrations in the organ bath.

### Experimental Protocols

#### Protocol 1: The role of endothelium in acetylcholine-induced vasodilation

To test whether ACh-induced responses were completely endothelium-dependent in preconstricted ophthalmic arteries of the C57BL/6J genotype, endothelium-intact and endothelium-denuded arteries were stimulated with ACh (10^−4^ M) and with the endothelium-independent NO donor, sodium nitroprusside (SNP, 10^−4^ M) to ensure that smooth muscle reactivity was not affected by endothelium removal^74^.

#### Protocol 2: Contribution of NO and cyclooxygenase (COX) metabolites to ACh- induced vasodilation

To assess the role of NO and prostanoids in mediating ophthalmic artery vasodilation, responses of arteries to cumulative application of ACh (10^−9^–10^−4^ M) were tested before and after incubation (30 min) with the non-isoform selective NOS inhibitor, L-NAME (10^−4^ M) or COX inhibitor, indomethacin (10^−5^ M). Similarly, responses of arteries to cumulative application of ACh were tested before and after treatment (30 min) with soluble guanylate cyclase (sGC) inhibitor, ODQ (10^−5^ M). Arteries were preconstricted with phenylephrine after the incubation with blockers.

#### Protocol 3: Contribution of EDHFs- mediated vasodilator response to ACh

To investigate the contribution of putative EDHFs to cholinergic vasodilation, responses of ophthalmic artery to ACh (10^−9^–10^−4^ M) were tested before and after 30 minutes of incubation with the following inhibitors alone or in combinations: 17-ODYA (10^−4^ M), a suicide substrate inhibitor of both ω- hydroxylation and epoxygenation of AA *via* the CYP450 pathway; baicalein (10^−5^ M), a specific inhibitor of 12/15-lipoxygenase (12/15-LOX); catalase (1000 units/ml), a hydrogen peroxide (H_2_O_2_) inhibitor; and 18α-GA (3 × 10^−5^ M), a gap junction uncoupler. Both L-NAME (10^−4^ M) and indomethacin (10^−5^ M) were present in the organ bath in addition to the inhibitors to prevent the formation of NO and prostanoids, respectively.

#### *Protocol 4: Contribution of calcium-activated potassium channels (K*_*Ca*_ *) and voltage-gated potassium channels (K*_*v*_ *) to ACh-induced vasodilation*

To characterize the K_Ca_ that mediate ACh-induced dilator reactivity, ophthalmic arteries were pre-treated with the combination of following agents: Apamin (10^−7^ M), a specific blocker of the small conductance K_Ca_ (SK_Ca_) and ChTX (10^−7^ M), an inhibitor of both intermediate conductance K_Ca_ (IK_Ca_) and big conductance K_Ca_ (BK_Ca_). Due to the limited specificity of ChTX, which also blocks some of the voltage-gated channels^34^, highly selective K_Ca_ blocker combinations were employed, as follows: IbTX (10^−7^ M), a selective BK_Ca_ blocker (Maxi K_Ca_) and TRAM-34 (10^−6^ M), a specific blocker of the IK_Ca_^60^. The role of specific *Shaker*-related type 1 K_v_ channels was evaluated employing blockers with varying sensitivity and specificity for the different K_v_ channels: MgTX (10^−8^ M), α- and β-DTX (5 × 10^−8^M) and MTX (5 × 10^−8^M).

#### Protocol 5: Contribution of potassium channels (Kir, and K_ATP_) and sodium-potassium pump (Na^+^/K^+−^ ATPase) to ACh- induced vasodilation

Depending on the channel, pump or enzyme targeted, vasodilatory responses of the vessels to ACh (10^−9^–10^−4^M) were tested before and after 30 minutes incubation with the following blockers: BaCl_2_ (10^−5^ M), an inward rectifier (Kir) channel blocker; ouabain (10^−4^ M) a Na^+^/K^+^- ATPase inhibitor and glibenclamide (10^−5^ M), a K_ATP_ channel inhibitor.

### Statistical analysis

Data are expressed as mean ± SEM, with *n* representing the number of animals per group. Changes in vascular responses to various reagents tested are presented as percentage of diameter change from the initial precontraction levels or the percent vasodilator responses as compared to maximal vasodilator response induced by ACh. Statistical comparisons of concentration- response curves were made using the two-way ANOVA for repeated measures followed by Bonferroni post-hoc test. Unpaired two-tailed *t*-test was used for single-dose responses. The level of significance α was set at 0.05. Graph Pad Prism 6 software (GraphPad Inc., San Diego, USA) was used for statistical analyses.

### Immunohistochemistry

To determine the localization of the K_v_1.6 channel in the mouse ophthalmic artery, segments of the ophthalmic artery were subjected to immunohistochemistry. The blood vessels were carefully isolated, rinsed in cold Krebs-Henseleit buffer and cryopreserved in Tissue-TEK OCT media (Sakura FineTek Europe, Alphen aan den Rijn, Netherlands) and immediately frozen at −20 °C in a freezer. Transverse cryosections of the arterial rings (8 μm thick) were thaw mounted onto Superfrost Plus slides (Thermo Scientific, Gerhard Menzel GmbH, Braunschweig, Germany), air-dried and stored at −20 °C until use. Prior to immunolabelling, the sections were fixed in 4% paraformaldehyde for 20 min, followed by permeabilization in PBS (0.05 M Na_2_HPO_4_, 0.14 M NaCl, pH 7.40) containing 0.1% Triton X-100 (TX). Sections were then blocked with PBS-TX containing 1% BSA and 10% normal goat serum for 30 min followed by overnight incubation with the primary antibody diluted at 1:50 at 4 °C. The rabbit polyclonal K_v_1.6 antibody (APC-003, Alomone Labs, Jerusalem, Israel) was generated against a glutathione S-transferase (GST) fusion protein corresponding to residues 463–530 of the rat K_v_1.6 protein. After overnight incubation, slides were rinsed in PBS and incubated with peroxidase conjugated polyclonal goat anti-rabbit IgG, H & L chain specific secondary antibody (Calbiochem, San Diego, CA, USA) at 1:200 for 1 h at room temperature. Negative control sections were incubated with blocking media and the secondary antibody. Sections were extensively rinsed to remove unbound antibody and the detection of antibody binding was carried out with Vector® NovaRED™ Substrate Kit for peroxidase (Vector Laboratories, Burlingame, CA, USA). Finally, slides were mounted and cover-slipped.

## Additional Information

**How to cite this article**: Manicam, C. *et al.* The Gatekeepers in the Mouse Ophthalmic Artery: Endothelium-Dependent Mechanisms of Cholinergic Vasodilation. *Sci. Rep.* **6**, 20322; doi: 10.1038/srep20322 (2016).

## Supplementary Material

Supplementary Figures S1-S11
