# Supplementary material for: The Gatekeepers in the Mouse Ophthalmic Artery: Endothelium-Dependent Mechanisms of Cholinergic Vasodilation
Source: Sci Rep. 2016 Feb 2;6:20322. doi: 10.1038/srep20322 (PMC4735817; doi:10.1038/srep20322)
Supplement: Supplementary Figures S1-S11 [file srep20322-s1.pdf]

SUPPLEMENTARY FIGURES

The Gatekeepers in the Mouse Ophthalmic Artery: Endothelium-Dependent Mechanisms of Cholinergic Vasodilation

Caroline Manicam[1*], Julia Staubitz[1], Christoph Brochhausen[2], Franz H. Grus[1], Norbert Pfeiffer[1], Adrian Gericke[1]

[1] Department of Ophthalmology, University Medical Center of the Johannes Gutenberg University Mainz, Mainz, Germany, [2] Institute of Pathology, University Medical Center, Johannes Gutenberg University Mainz, Germany.

[*]**Corresponding author:**

Dr. Caroline Manicam

Department of Ophthalmology, University Medical Center of the Johannes Gutenberg University Mainz, Langenbeckstr. 1, 55131 Mainz, Germany.

E-mail: cmjc_82@yahoo.com

Tel: +49(0) 6131 17 3330

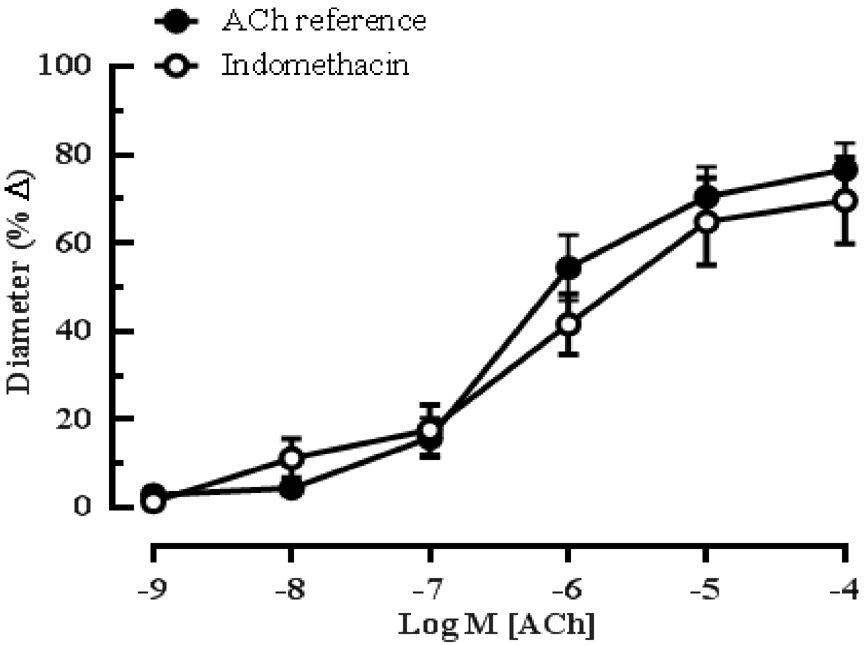

Supplementary Figure S1

Effect of COX inhibition on the vasodilatory responses of ophthalmic artery from wild-type mice with intact endothelium.

Responses of ophthalmic artery to ACh remained unaltered after treatment with the COX inhibitor, indomethacin (10^{-5} M).Values are expressed as mean ± s.e.m (n =9 per group).

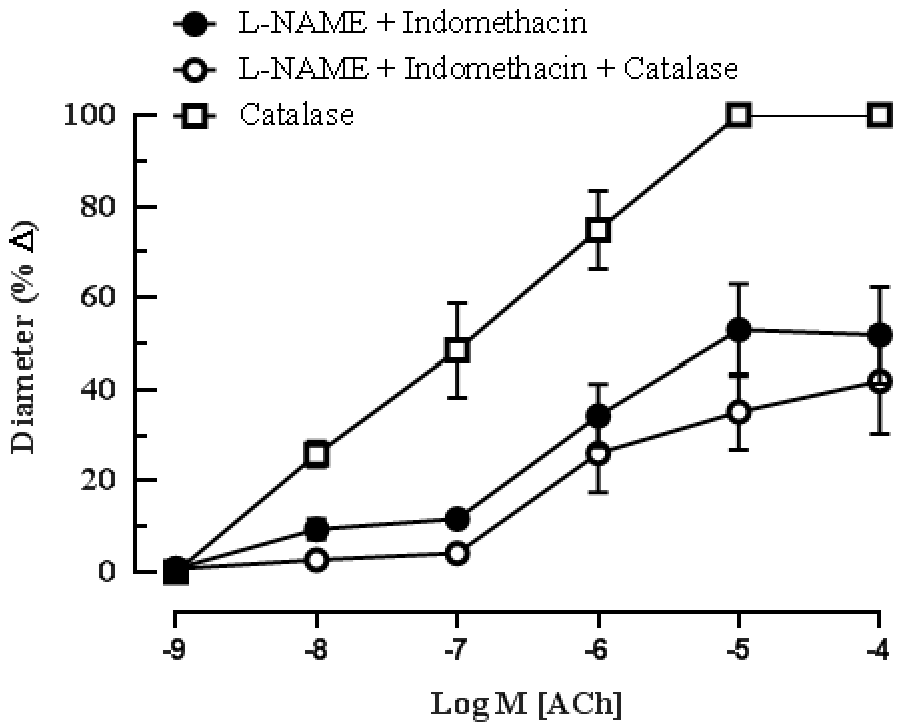

Supplementary Figure S2

Catalase elicited negligible inhibitory effect on ACh-induced vasodilation responses. Blocking of H_2O_2 with catalase (1000 units/ml) in the mouse ophthalmic artery did not cause significant attenuation of the vasodilation, when applied either alone or in combination with L-NAME and indomethacin. Values are expressed as mean ± s.e.m (n =5 per group).

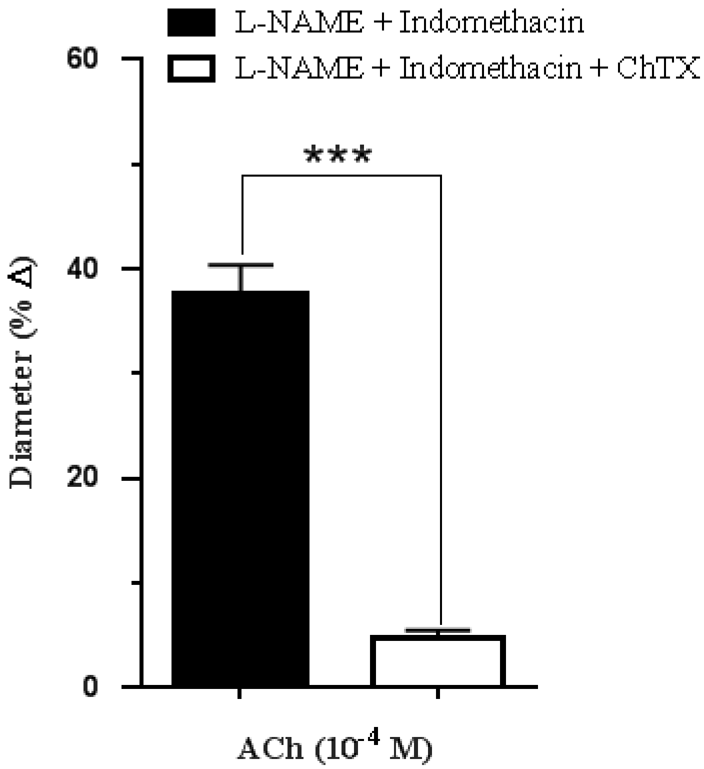

Supplementary Figure S3

Inhibition of vasodilation with ChTX in combination with L-NAME+ Indomethacin almost completely abolished vasodilation. The use of ChTX in the presence of both NOS and COX blockers significantly inhibited the vasodilation to ACh (4.69 ± 0.79 %) in comparison to L-NAME and Indomethacin (37.7 ± 2.63 %). This finding is consistent with the hypothesis that the ChTX-sensitive channel(s) are actively involved in mediating the NOS- independent vasodilatory responses. Values are expressed as mean ± s.e.m (n =7 per group ****P* <0.001, L-NAME and Indomethacin versus L-NAME and Indomethacin and ChTX).

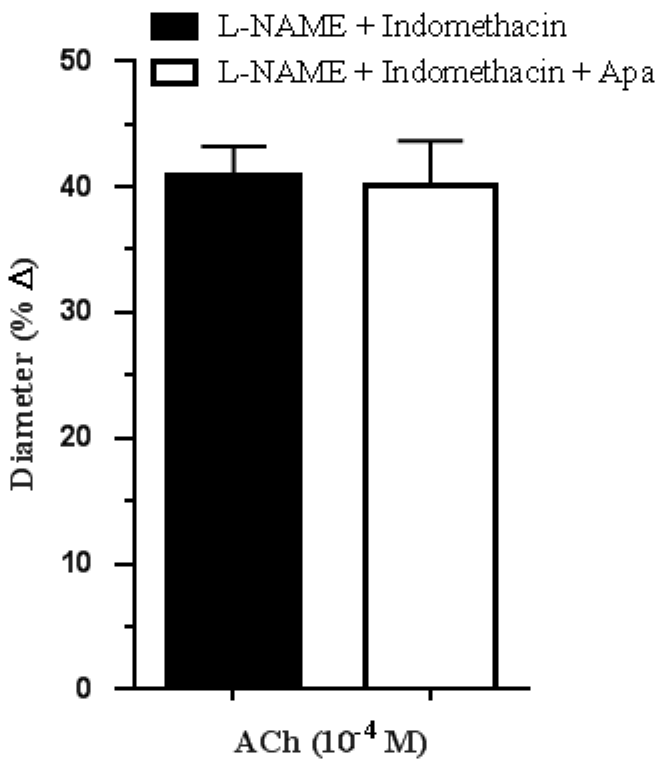

Supplementary Figure S4

Apamin-sensitive SK_{Ca} channels are not involved in the vasodilatory responses in the mouse ophthalmic artery. The specific blocking of the SK_{Ca} channel elicited no inhibitory effects on the vasodilation, indicating null involvement of this channel subtype in mediating the efflux of K^+ for hyperpolarization to occur in the ophthalmic artery. It is also hypothesized that this channel subtype may not be expressed in the mouse ophthalmic artery. Values are expressed as mean ± s.e.m (n =10 per group).

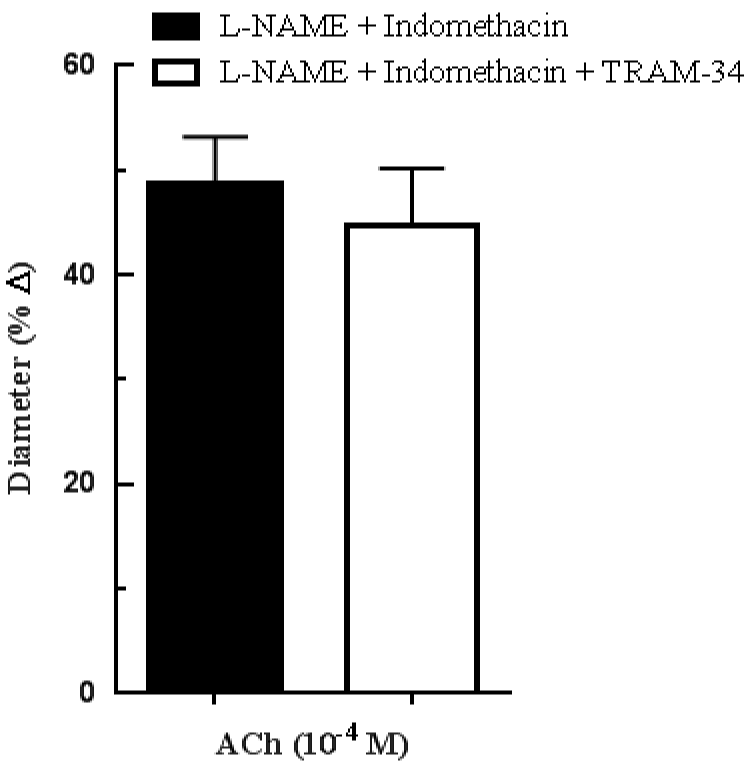

Supplementary Figure S5

TRAM-34-sensitive IK_{Ca} channels are not involved in the vasodilatory responses in the mouse ophthalmic artery. The specific blocking of the IK_{Ca} channel with TRAM-34 evoked no inhibitory effects on the vasodilation, indicating null involvement of this channel subtype in mediating the efflux of K^+ for hyperpolarization to occur in the ophthalmic artery. It is also highly probable that this channel subtype is not expressed in the mouse ophthalmic artery. Values are expressed as mean ± s.e.m (n =7 per group).

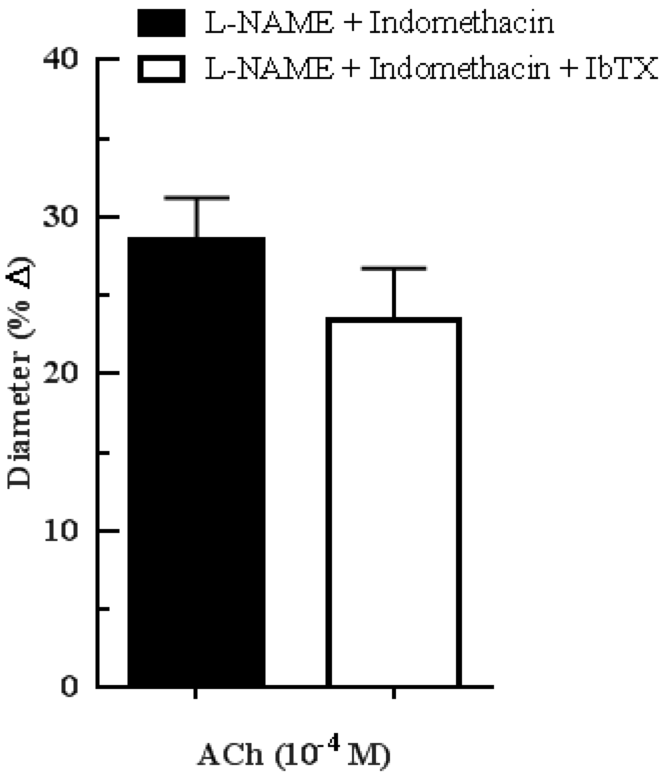

Supplementary Figure S6

IbTX-sensitive BK_{Ca} channels are not involved in the vasodilatory responses in the mouse ophthalmic artery. The specific blocking of the BK_{Ca} channel with IbTX evoked no inhibitory effects on the vasodilation, indicating null involvement of this channel subtype in mediating the efflux of K^+ for hyperpolarization to occur in the ophthalmic artery. There is a high probability that this channel subtype is not expressed in the mouse ophthalmic artery. Values are expressed as mean ± s.e.m (n =8 per group).

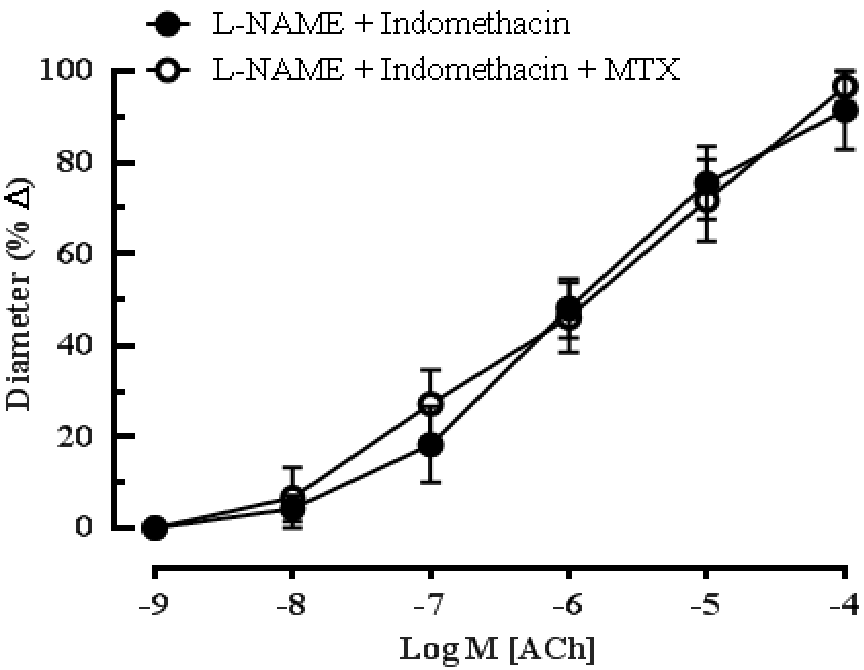

Supplementary Figure S7

Inhibitory effect of MTX on the vasodilatory responses of ophthalmic artery from wild-type mice.

The blocking of the artery with MTX, which inhibits with high specificity the K_v 1.2 channel showed no inhibitory effects on the ACh-mediated vasodilation.Values are expressed as mean ± s.e.m (n =5 per group).

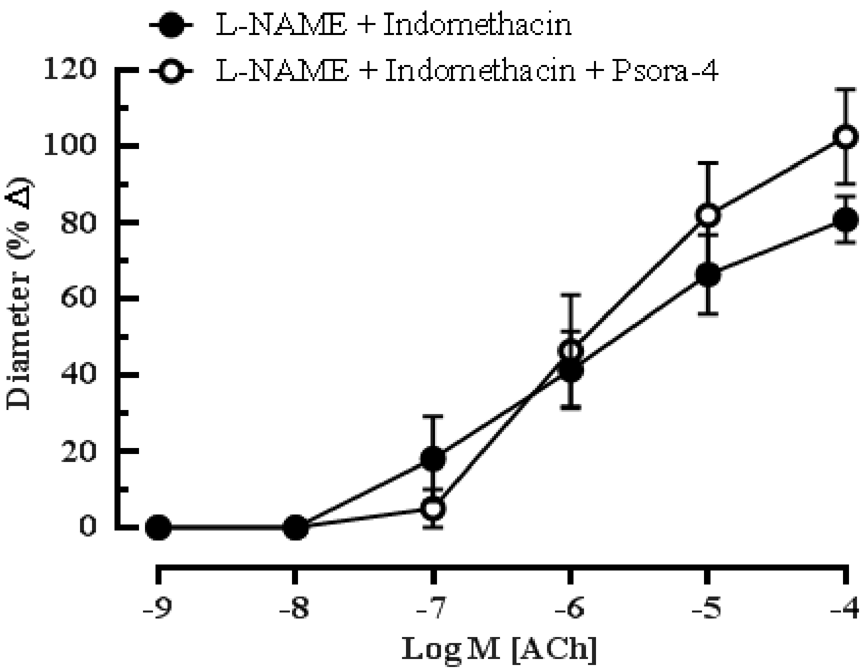

Supplementary Figure S8

Effect of psora-4 inhibition on the vasodilatory responses of ophthalmic artery from wild-type mice.

The blocking of the artery with psora-4, which inhibits with high specificity the K_v 1.3 channel showed no inhibitory effects on the ACh-mediated vasodilation.Values are expressed as mean ± s.e.m (n =5 per group).

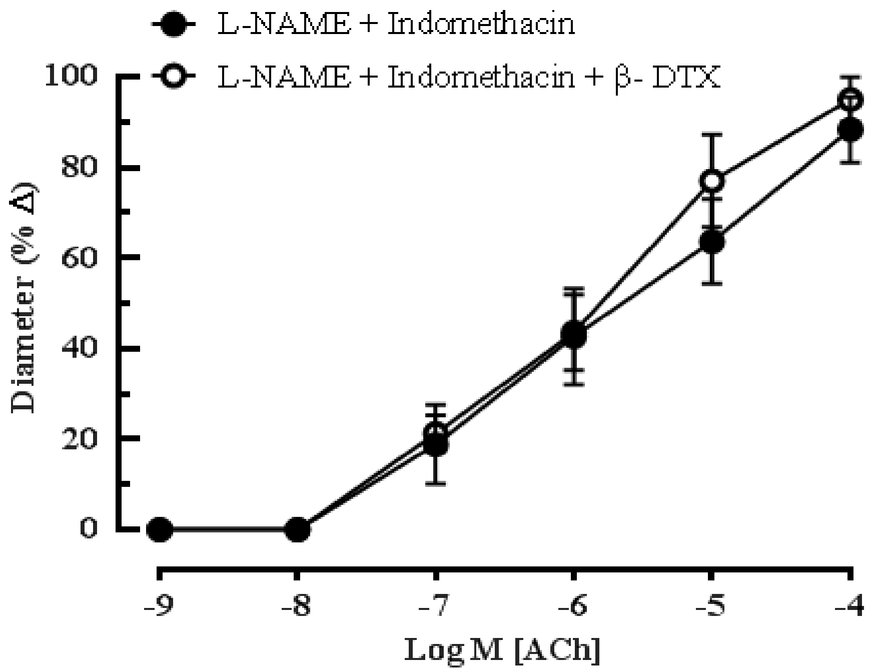

Supplementary Figure S9

Effect of β-DTX inhibition on the vasodilatory responses of ophthalmic artery from wild-type mice.

The blocking of the artery with β-DTX, which inhibits both K_v 1.1 and K_v 1.2 channels showed no inhibitory effects on the ACh-mediated vasodilation.Values are expressed as mean ± s.e.m (n =5 per group).

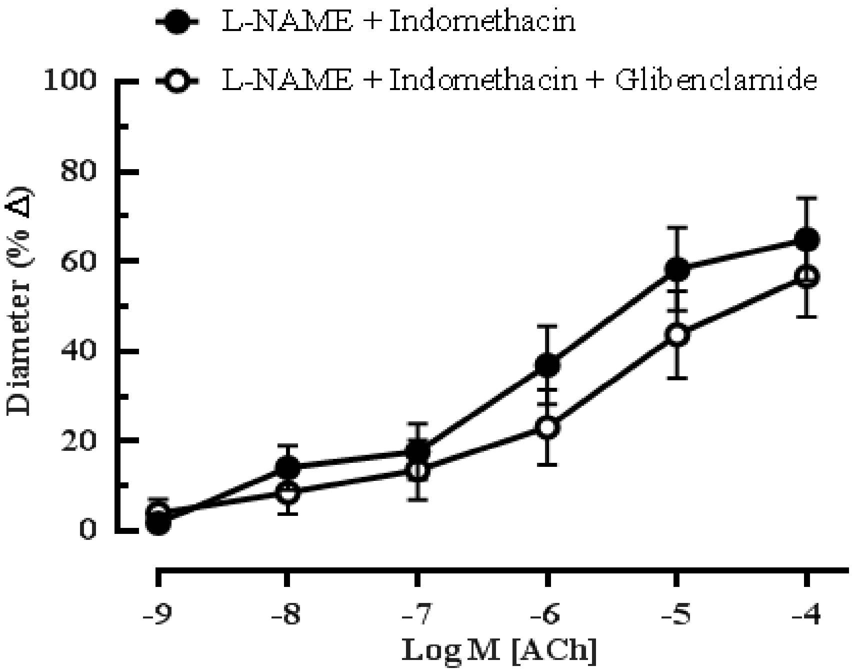

Supplementary Figure S10

Effect of K_{ATP} channel inhibition on ophthalmic artery vasodilation.

The incubation of the vessels with glibenclamide, which blocks the K_{ATP} channel showed non-significant inhibitory effects on the ACh-mediated vasodilation.Values are expressed as mean ± s.e.m (n =6 per group).

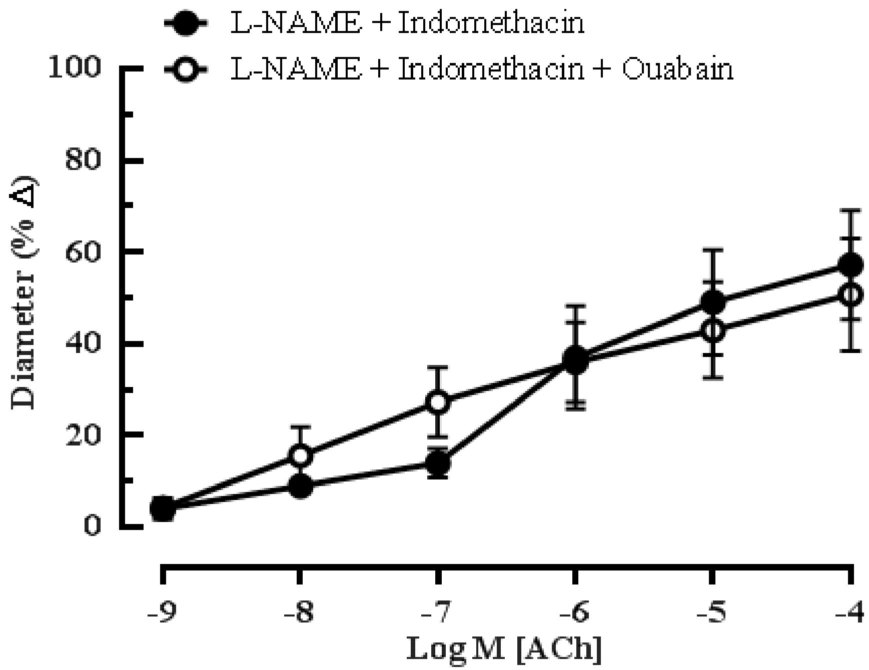

Supplementary Figure S11

Effect of Na+/K+-ATPase inhibition on ophthalmic artery vasodilation.

The incubation of the vessels with ouabain, which blocks the Na^+/K^+-ATPase showed no significant inhibitory effects on the ACh-mediated vasodilation.Values are expressed as mean ± s.e.m (n =5 per group).
